# Development of Dendritic Cells from GM-CSF^-/-^ Mice *in* *vitro* : GM-CSF Enhances Production and Survival of Cells

**DOI:** 10.1155/2001/68024

**Published:** 2001

**Authors:** Keping Ni, Helen C. O'Neill

**Affiliations:** Division of Biochemistry & Molecular Biology School of Life Sciences Australian National University Canberra ACT 0200 Australia

**Keywords:** dendritic cells, long term culture, stroma, haemopoiesis, development

## Abstract

The production of dendritic cells (DC) from haemopoietic progenitors maintained in long term stroma-dependent cultures (LTC) of spleen or bone marrow (BM) occurs independently of added granulocyte/macrophage colony stimulating factor (GM-CSF). The possibility that cultures depend on endogenous GM-CSF produced in low levels was tested by attempting to generate LTC from spleen and BM of GM-CSF^-/-^ mice. Multiple cultures from GM-CSF^-/-^ and wild type mice were established and compared for cell production. GM-CSF^-/-^ LTC developed more slowly, but by 16 weeks produced cells resembling DC in numbers comparable to wild type cultures. LTC maintained distinct populations of small and large cells, the latter resembling DC. Cells collected from GM-CSF^-/-^ LTC were capable antigen presenting cells (APC) for T cell stimulation and morphologically resembled DC. Large cells expressed the CD11b, CD11c, CD86, 33D1 and Dec-205 markers of DC. Addition of GM-CSF to GM-CSF^-/-^ LTC increased the proportion of large, mature DC present in culture. Stromal cells from GM-CSF^-/-^ LTC could support the differentiation of DC from early progenitors maintained in LTC without addition of GM-CSF. However, GM-CSF is not a critical factor in the *in* *vitro* generation of DC from progenitors. It can, however, substitute for stromal cells in increasing the survival of mature DC.


*Developmental Immunology*, 2001, Vol. 8(2), pp. 133-146
Reprints available directly from the publisher
Photocopying permitted by license only

© 2001 OPA (Overseas Publishers Association)
N.V. Published by license under
the Harwood Academic Publishers imprint,
part of The Gordon and Breach Publishing Group,
a member of the Taylor and Francis Group

### Development of Dendritic Cells from GM-CSF$^{-/-}$ Mice *in vitro* : GM-CSF Enhances Production and Survival of Cells

KEPING NI and HELEN C. O'NEILL*

*Division of Biochemistry & Molecular Biology, School of Life Sciences, Australian National University, Canberra, AUSTRALIA*

The production of dendritic cells (DC) from haemopoietic progenitors maintained in long term stroma-dependent cultures (LTC) of spleen or bone marrow (BM) occurs independently of added granulocyte/macrophage colony stimulating factor (GM-CSF). The possibility that cultures depend on endogenous GM-CSF produced in low levels was tested by attempting to generate LTC from spleen and BM of GM-CSF$^{-/-}$ mice. Multiple cultures from GM-CSF$^{-/-}$ and wild type mice were established and compared for cell production. GM-CSF$^{-/-}$ LTC developed more slowly, but by 16 weeks produced cells resembling DC in numbers comparable to wild type cultures. LTC maintained distinct populations of small and large cells, the latter resembling DC. Cells collected from GM-CSF$^{-/-}$ LTC were capable antigen presenting cells (APC) for T cell stimulation and morphologically resembled DC. Large cells expressed the CD11b, CD11c, CD86, 33D1 and Dec-205 markers of DC. Addition of GM-CSF to GM-CSF$^{-/-}$ LTC increased the proportion of large, mature DC present in culture. Stromal cells from GM-CSF$^{-/-}$ LTC could support the differentiation of DC from early progenitors maintained in LTC without addition of GM-CSF. However, GM-CSF is not a critical factor in the *in vitro* generation of DC from progenitors. It can, however, substitute for stromal cells in increasing the survival of mature DC.

*Keywords:* dendritic cells, long term culture, stroma, haemopoiesis, development

### INTRODUCTION

Dendritic cells (DC) are central to immune response development, being the primary antigen presenting cells (APC) capable of stimulating naïve T cells. Attempts to study DC development and to identify genes and proteins which regulate cell differentiation and function have been impeded by the scarcity of DC in tissues and the difficulties associated with their isolation.

There is currently evidence for at least three distinct DC lineages, including Langerhans cells in skin, myeloid DC and lymphoid DC (Shortman & Caux, 1997). It is believed that GM-CSF is an essential factor for development of DC of the myeloid lineage. The importance of GM-CSF has been demonstrated in many independent studies using different starting cell populations including CD34$^+$ BM progenitors, peripheral blood monocytes and DC precursors isolated by various means from a number of lymphoid

* Address for correspondence: Dr HC O'Neill, Division of Biochemistry & Molecular Biology, School of Life Sciences, Australian National University, Canberra, ACT 0200 AUSTRALIA. PH: 61 2 6125 4720; FAX: 61 2 6125 0313.

sites including spleen, lymph node and bone marrow (BM) (reviewed by Caux & Banchereau, 1996). For example, GM-CSF, Interleukin(IL)-4 and TNF-α are used commonly to induce DC production from blood monocytes for use in immunotherapy. Similar combinations of GM-CSF, IL-4 and stem cell factor (SCF) can be used to expand DC out of BM or cord blood.

Despite the common usage of GM-CSF in DC culture, mutant mice deficient in production of either GM-CSF or its receptor still produce nearly normal numbers of DC (Vremec et al., 1997). Similarly, GM-CSF transgenic mice or mice receiving an infusion of GM-CSF, do not have increased numbers of DC in most lymphoid sites except in lymph node and peritoneal cavity (Maraskovsky et al., 1996; Vremec et al., 1997). These results are consistent with other evidence that development of lymphoid DC from thymic precursors or pro-B cells can occur independently of GM-CSF (Bjork & Kincade, 1998; Saunders et al., 1996).

A long term culture (LTC) system for production of murine DC has been developed which generates a continuous supply of immature DC from haemopoeitic precursors maintained within the culture without the addition of GM-CSF (Ni & O'Neill; 1997,1998,1999; O'Neill et al., 1999a). Continuous production of cells depends on the presence of a stromal cell layer of endothelial and fibroblastic cells. A constant population of cells is released into the culture supernatent and can be readily collected for analysis. Cells produced in LTC have a cell surface phenotype resembling DC with high levels of CD11b, CD11c, 33D1 and CD80/CD86 (Ni & O'Neill, 1997; Ni & O'Neill, 2000). Their cell surface phenotype is more consistent with myeloid DC since the great majority of cells are CD8α$^-$ and Dec-205$^-$.

LTC-DC maintain the functional characteristics of DC, being endocytic (Ni & O'Neill, 1997) and highly potent APC (O'Neill et al., 1999a). They are migratory and can induce a protective anti-tumour immune response when pulsed with tumour membranes prior to adoptive transfer into mice (O'Neill et al., 1999b). The majority of CD11c$^+$ cells produced in culture do not express the CD8α marker (Ni & O'Neill, 2000) which has been used to identify murine lymphoid DC in thymus (Saunders et al., 1997), spleen (Leenan et al., 1998) and lymph node (Salomon et al., 1998). This result is consistent with LTC producing myeloid DC. However, development of DC in LTC depends on the presence of stromal cells and occurs independently of added GM-CSF, which is more consistent with lymphoid DC (Ni & O'Neill; 1997, 1998). Growth factors which support DC production from progenitors remain undefined, but DC production occurs independently of factors known to affect DC colony formation including GM-CSF, IL-3, IL-4, IL-6, TNF-α, SCF, IL-7 and IL-1 (Wilson et al., 2000 a; Ni & O'Neill, 1997).

Since DC of different lineages may be functionally distinct it is important to determine the lineage characteristics of LTC-DC and the soluble factors and signaling events essential to their development. The hypothesis that LTC-DC production is driven by low levels of endogenously produced GM-CSF has been tested by attempting to generate LTC producing DC from spleen and BM of GM-CSF$^{-/-}$ mice. Cultures producing DC can be generated independently of GM-CSF in knockout mice. Furthermore, GM-CSF can substitute *in vitro* for other stroma-derived factors in supporting the survival of cells already committed to the DC lineage.

### RESULTS

### Production of LTC-DC from spleen and BM occurs independently of GM-CSF

Spleen and BM from GM-CSF$^{-/-}$ [129 × C57BL/6J] $F_1$mice and syngeneic wild type (WT) mice were compared for production of LTC supporting haemopoiesis. At least 6 spleen LTC were established from individual mice of each strain and development of cells followed over time. Success rate in establishment of LTC was similar for both strains although stroma development was slower in GM-CSF$^{-/-}$ LTC. After 10 days, spleen LTC from GM-CSF$^{-/-}$ mice had less endothelial cell growth and produced fewer non-adherent cells than LTC from WT spleen (Fig. 1).

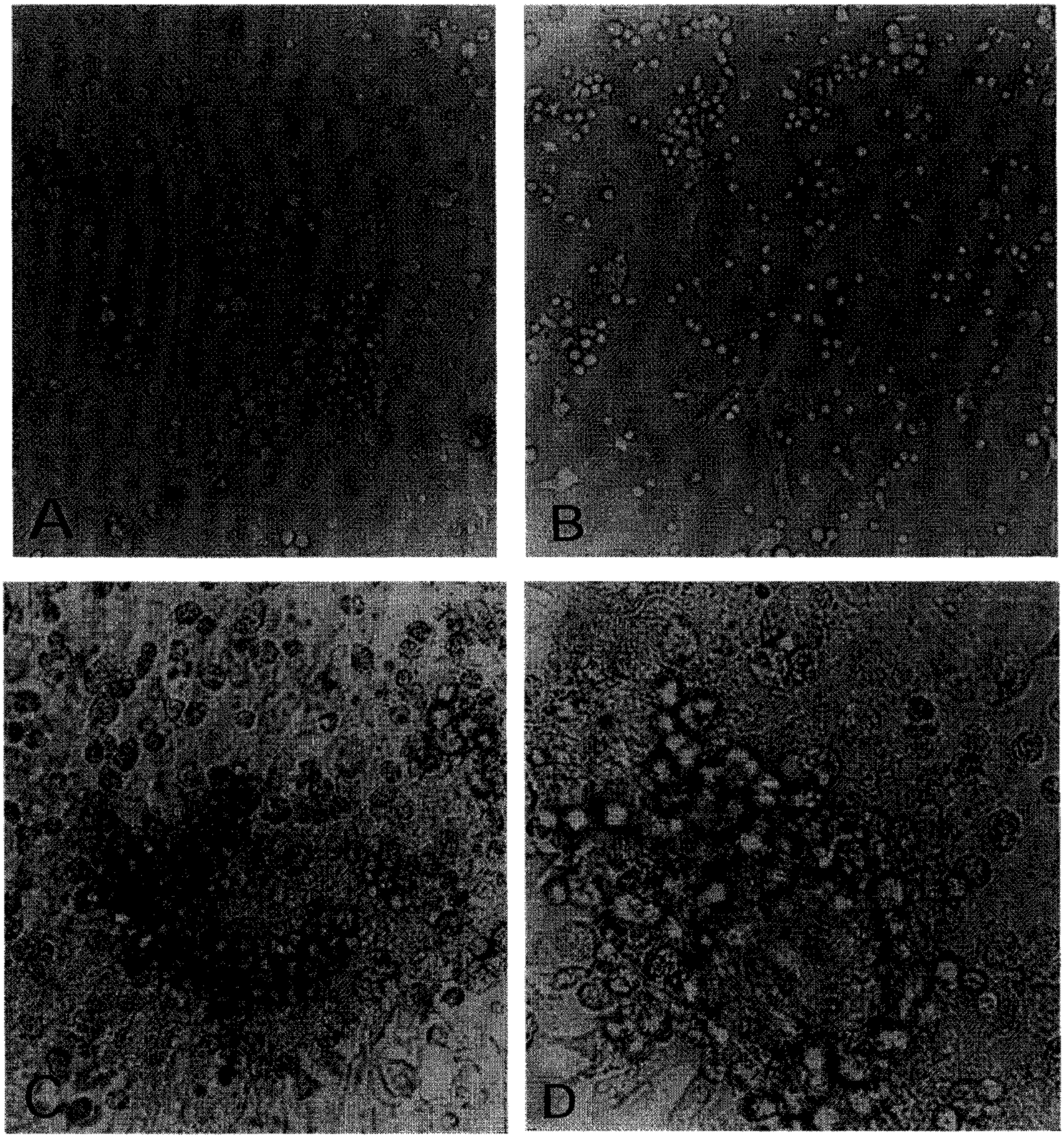

FIGURE 1 Development of DC in spleen LTC from GM-CSF$^{-/-}$ mice. LTC from GM-CSF$^{-/-}$ (A,C) and wild type (WT) (B,D) littermate control mice are shown at 10 days (A,B) and 28 days (C,D). Differences in extent of stroma development are shown at 10 days. Haemopoietic cells forming on top of stroma is shown in LTC from GM-CSF$^{-/-}$ and wild type (WT) littermate control mice at 4 weeks when DC with typical morphology are released into the supernatent. Magnification : 200x (A, B, D), 320x (C)

Over the same period, WT spleen LTC supported haemopoiesis of cells earlier than did GM-CSF$^{-/-}$ LTC. By 4 weeks there was more similarity between cultures. Clear foci were present in both GM-CSF$^{-/-}$ and WT cultures on well developed stroma containing both fibroblasts and endothelial cells (Fig. 1). DC

were detectable as non-adherent cells present in the medium of both GM-CSF$^{-/-}$ and WT LTC after 4 weeks of culture.

Interdigitating DC were microscopically detectable amongst freshly isolated BM cells of GM-CSF$^{-/-}$mice suggesting that development of DC *in vivo* can occur independently of GM-CSF (data not shown). Stroma was well developed by 10 days in BM LTC from both GM-CSF$^{-/-}$and WT mice. At this stage, fewer haemopoietic cells were present in GM-CSF$^{-/-}$than WT cultures (Fig. 2), consistent with absence of GM-CSF which supports early BM haemopoiesis. By 4 weeks, stroma was well developed in all LTC, with clear evidence of DC amongst the non-adherent cells (Fig. 2).

LTC derived from spleen and BM of both GM-CSF$^{-/-}$ and WT mice were easily maintained for 16 weeks. Cell production was monitored continuously over that time by collection of an aliquot of non-adherent cells. The presence of two clear subsets of large and small cells, characteristic of LTC (Wilson et al., 2000 b), was assessed by Forward and Side Scatter analysis on the FACS. By comparison, the well established LTC-X1 line maintains a constant high production (87%) of large DC amongst the non-adherent cell population (Fig. 3). The percentage of large cells produced by GM-CSF$^{-/-}$ spleen LTC increased steadily over 11 weeks to reach levels of 30–40% of non-adherent cells (Fig. 3). The percentage of large cells produced in GM-CSF$^{-/-}$ BM LTC was much lower, reaching levels of only ~10% after 11 weeks. By 14 weeks, GM-CSF$^{-/-}$ spleen LTC contained 35% large cells while WT spleen LTC had ~48% large cells. In line with previous findings on BM LTC which are slower producers of DC (Ni and O'Neill, 1997), the large cell population in both GM-CSF$^{-/-}$ and WT BM LTC represented only ~20% of cells. A secondary 14-week culture of GM-CSF$^{-/-}$ BM on LTC-X1 stroma (Ni & O'Neill, 1999) produced ~30% large DC.

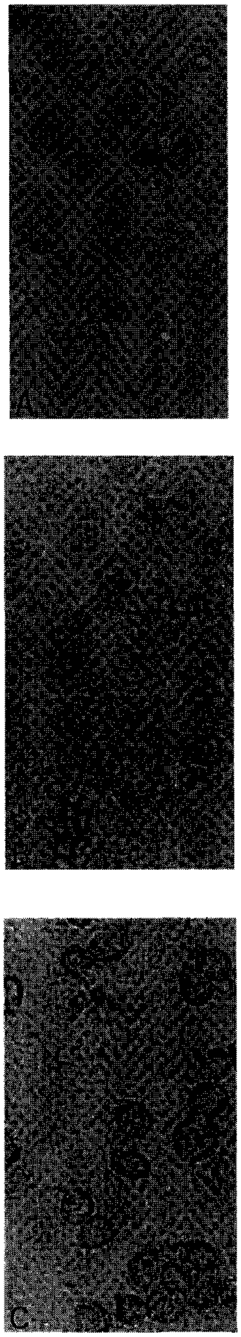

FIGURE 2 Development of DC in BM LTC from GM-CSF$^{-/-}$ mice. BM LTC derived from GM-CSF$^{-/-}$ is shown at 10 days and 4 weeks (A,C). BM LTC from wild type (WT) control mice is shown at 10 days (B). Well developed stroma was observed by day 10 in all cultures but haemopoiesis occurred only in WT cultures (B). Dendritic-like cells were present in all cultures of WT and GM-CSF$^{-/-}$ mice by 4 weeks (C)

### Phenotypic identification of DC produced in GM-CSF$^{-/-}$ LTC

LTC were monitored over time for production of cells representing different haemopoietic lineages using

FACS analysis and antibodies specific for markers expressed by myeloid, dendritic and lymphoid cells. Multiple cultures were analysed for up to 14 weeks to estimate the proportion of different cell types present in the non-adherent cell subset produced during the early stages of LTC development. Over the first 3 to 7 weeks, cells produced comprised a mixture of small cells with low Forward and Side Scatter, subpopulations of which stained positively for CD11b (myeloid/DC) and 33D1 (DC) (all data not shown). Clear subpopulations of small B220$^+$ B cells and Thy-1$^+$ T cells were detectable in spleen LTC. By 11 weeks, these subsets had virtually disappeared. Similar results were obtained for all cultures but representative results obtained for just one GM-CSF$^{-/-}$ spleen LTC, 8GS1.4, are given. FACS profiles of cell surface marker on non-adherent cells collected from 8GS1.4 at 11 to 14 weeks after initiation of culture are shown in Fig. 4 along with profiles of the established LTC-X1 cell line. Cells with high Side Scatter in LTC-X1 stain strongly for CD11b, 33D1, CD11c and F4/80 with subsets of cells staining for MHCII and Dec-205 (Fig. 4 & Wilson et al., 2000 b). There are no cells present which express Thy-1 and B220 which mark lymphoid cells. No Gr-1$^+$ cells are produced by LTC-X1 (data not shown).

Cultures of 8GS1.4 showed clear development of very large cells with high FSC/SSC. Cells with high SSC stained positively for CD11b, CD11c and F4/80 in numbers comparable with LTC-X1 (Fig. 4). A high proportion (45%) of cells expressed Dec-205, a DC marker, and MHC Class II (71%), which is expressed by DC at different stages during development. These two markers are expressed by a smaller subset of LTC-X1. The presence of a small population of DC expressing high levels of MHCII in LTC-X1 has been associated with a predominance of immature DC with low expression of MHCII (Ni & O'Neill, 2000). DC produced in GM-CSF$^{-/-}$ LTC at 14 weeks differ from LTC-X1 derived DC in terms of MHCII expression. High expression of cell surface MHCII on DC has been associated with very early DC precursors and with mature DC capable of T cell stimulation (Pierre et al., 1997). By 14 weeks, cultures contained a large subpopulation of Thy-1$^+$ cells (~50%) with high SSC distinguishable from a 4% population of small Thy-1$^+$T cells. The significance of Thy-1 expression is not yet clear, but aberrant expression could be related to culture conditions.

Although cells produced in GM-CSF$^{-/-}$ LTC do not uniformly express DC markers until ~11 weeks of culture, cells present in the early stages of culture are very effective APC in an MLR. In Fig. 5, non-adherent cells collected from the GM-CSF$^{-/-}$ spleen LTC 8FZ8.1 after 8 weeks, can stimulate proliferation of syngeneic (C57BL/6J) and semi-allogeneic (B10.A(2R)) spleen responders with up to 25-fold increase in proliferation above background. In contrast, LTC-X3 cells are more effective stimulators of lymphocytes of B10.A(2R) and allogeneic BALB/c and C57BL/6J responders. These effects can be achieved with very small numbers of APC, e.g. <100 APC per well. Functional immunostimulatory DC are produced in GM-CSF$^{-/-}$ spleen LTC as early as 8 weeks before clear populations of DC are identifiable by marker expression.

Across all LTC there was consistent, slow outgrowth of DC in spleen and BM LTC of GM-CSF$^{-/-}$mice as other cell subsets disappeared from culture. The outgrowth of DC in early cultures was, however, slower than in LTC derived from WT or other non-mutant strains of mice (Ni & O'Neill; 1997,1998). Cell production was maintained even after 16 weeks for 8GS1.4, with 41% of cells representing large DC with high FSC. The proportion of CD11c$^+$ cells had increased to 95% of large cells cells. Only 21% of the CD11c$^+$ cells coexpressed MHCII, which is more consistent with marker distribution on LTC-X1 cells and the predominance of immature DC in LTC (Fig. 6). Amongst the small cell population, 88% of cells were CD11c$^+$ with 49% of cells expressing low levels of MHCII.

### Effect of addition of GM-CSF to GM-CSF$^{-/-}$ LTC

GM-CSF is not a critical factor in the development of DC from progenitors maintained in LTC. To test the alternative hypothesis that GM-CSF may act at a later stage during development to support replication, development or maintenance of DC precursors,

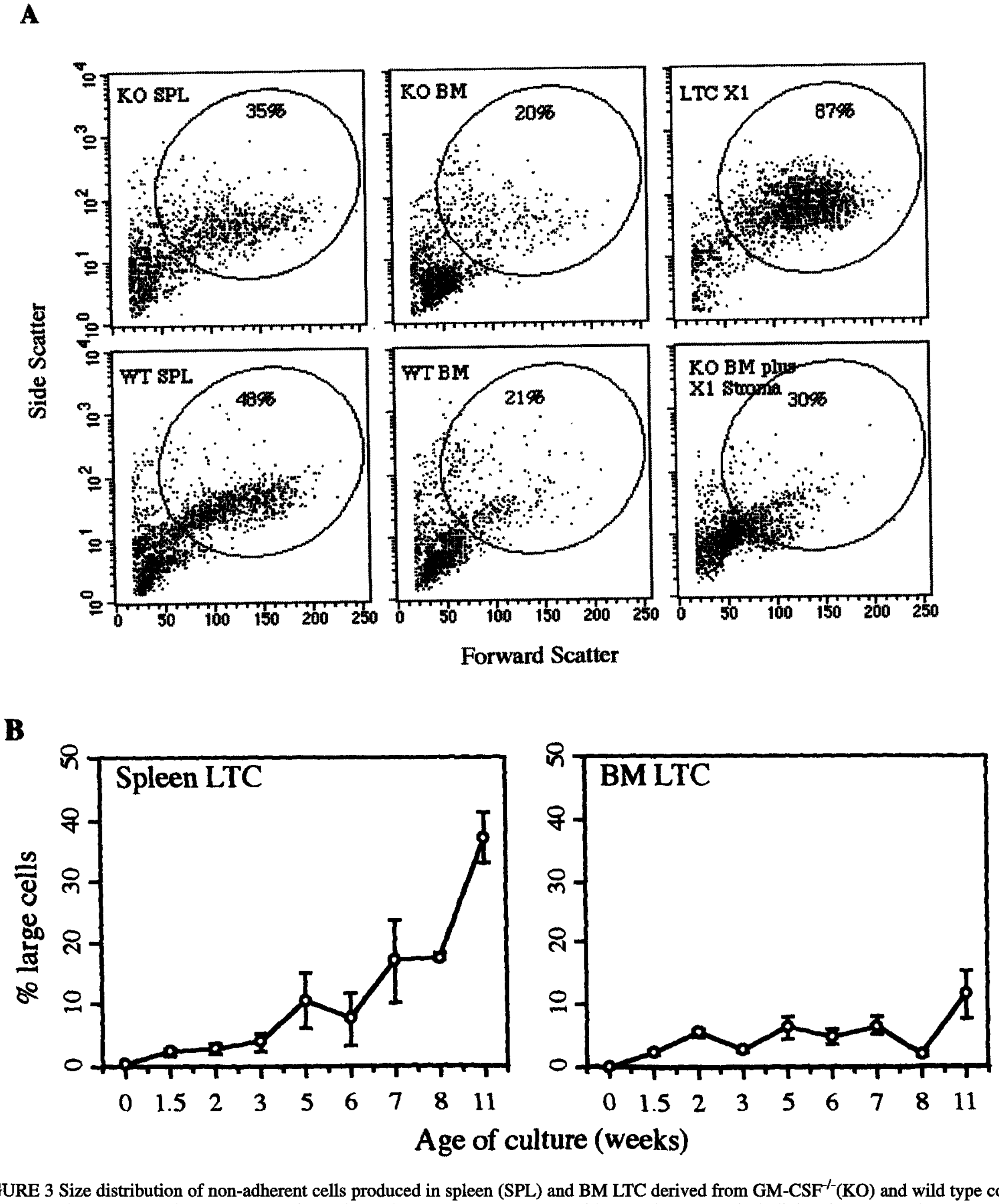

FIGURE 3 Size distribution of non-adherent cells produced in spleen (SPL) and BM LTC derived from GM-CSF$^{-/-}$(KO) and wild type control (WT) mice. A.) Data from representative cultures indicate the percentage of large cells present in individual cultures. Gates were set according to the distribution of large cells present in the well established LTC-X1 cell line. Development of KO BM on LTC-X1 stroma (secondary LTC) was also compared. B.) The production of large cells by spleen and BM LTC of GM-CSF$^{-/-}$ mice was followed over 11 weeks (mean±SE of eleven cultures)

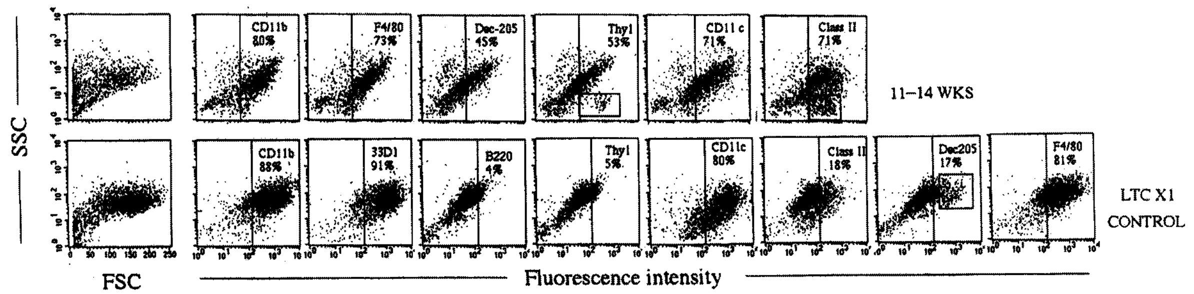

FIGURE 4 Cell surface antigen expression on cells produced in LTC 8GS1.4 derived from GM-CSF$^{-/-}$ mice at 11 to 14 weeks after establishment of cultures. Non-adherent cells were collected from LTC and stained using specific fluorochrome-labelled antibodies. The production of large DC is indicated by the population of cells with high FSC and SSC (left column). Marker expression is indicated in plots of SSC versus Fluorescence. Percentage of cells staining with specific fluorescent antibody was estimated using isotype control antibodies to set gates for positive staining. Staining of DC produced by the established LTC-X1 line is shown in the bottom row for comparison. Gates are included in some plots to indicate subsets which stain positively

GM-CSF was added into the established 8GS4.1 GM-CSF$^{-/-}$ spleen-derived LTC at 16 weeks. The effectof GM-CSF on production of DC was monitored by FACS analysis over 12 days. GM-CSF was maintained at 300 U/ml for 12 days with a medium change every 3 days and the size and marker expression of cells produced was monitored by removal of an aliquot of non-adherent cells.

Initially, addition of GM-CSF resulted in an increase in the number of large non-adherent cells present in culture. By 3 days there was a notable increase in the % large cells over the starting population (Day 0). The culture composition changed from 41% to 76% large cells and a higher proportion of large cells was maintained over the 12 days of the experiment (Fig. 6). GM-CSF could be acting to increase the number of small or precursor cells which develop into large cells or enhance the number of large cells. There was a transient reduction in the level of expression of CD11c on both the large and small cells at 3 days coincident with this change in cell production (Fig. 6). However, by 12 days, the proportion of large cells which are CD11c$^+$ increased to 73%, with 65% of small cells also CD11c$^+$. Small subpopulations of CD11c$^+$MHCII$^+$cells were also detectable. Similar changes were also observed in 3 other LTC after addition of GM-CSF (all data not shown). By 12 days after GM-CSF addition, distribution of CD11c$^+$ or MHCII$^+$ cells amongst the small and large cell subsets resembled that of the starting cell population. The DC phenotype of cells was also confirmed by the high expression of CD86 on 96% of the large cells and on ~90% of the small cells. This was measured at 6 and 12 days after exposure to GM-CSF (Fig. 6). CD86 is consistently expressed by >90% of large LTC-X1 cells and by ~90% of small cells and has been consistently expressed on LTC-X1 cells over ~7 years (Fig. 6). In similar experiments using LTC from norml mice, GM-CSF has been shown to expand the number of large DC produced (Ni & O'Neill, data not shown). However, continuous addition of GM-CSF to cultures reduces the small cell population over time and DC production in LTC ceases.

### GM-CSF acts as a maintenance factor for DC

The effects of GM-CSF on DC versus their progenitors was determined by assessing the type of cells collected from LTC which survive and proliferate to form colonies in semi-solid agar colony assays. Assays involved non-adherent cells collected from GM-CSF$^{-/-}$ spleen LTC. These assays have been performed previously to quantitate the production of DC colonies from non-adherent cells generated in LTC (Wilson et al., 2000 a). GM-CSF supported the for-

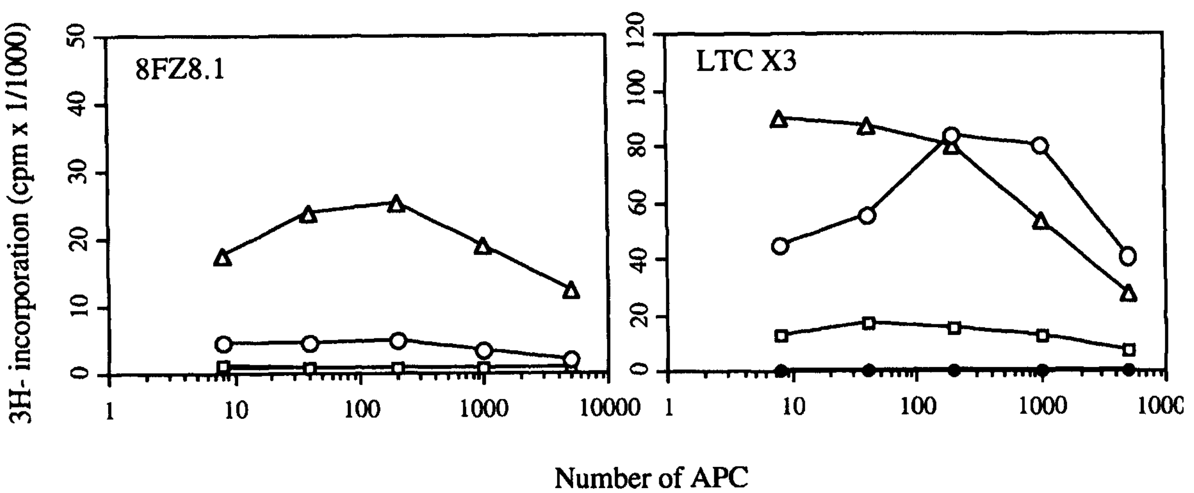

FIGURE 5 Induction of MLR by DC produced after 8 weeks by spleen LTC derived from GM-CSF$^{-/-}$ mice (8FZ8.1). Responders were $10^5$ spleen mononuclear cells from different strains of mice: BALB/c(□), C57BL/6J(O), and B10.A(2R)(Δ). These were cultured for three days in an MLR with varying numbers of non-adherent APC derived from 8FZ8.1 or from control B10.A(2R)-derived LTC X3. Proliferation was measured by incorporation of $^3$H-T in the final 6 hours of culture. Controls included non-adherent cells from 8FZ8.1 alone (●). Responders in the absence of 8FZ8.1 gave backgrounds of <800 cpm mation of a small number of ~30 colonies of cells with short membrane extensions (Type II; Table I). Colonies representative of other haemopoietic cell types or undifferentiated cells, were not detected in these experiments. This confirms the production of only DC/DC progenitors in LTC. A smaller number of similar colonies also formed when cultures were supplemented with stromal cells ($10^4$) collected from GM-CSF$^{-/-}$ spleen LTC (Table 1). Not all progenitors produced in LTC are GM-CSF-dependent but a small, significant number, perhaps representing a different lineage, do respond to GM-CSF. Conditioned medium alone from LTC derived from either GM-CSF$^{-/-}$ or WT spleen did not provide equivalent growth support in these experiments.

TABLE I Conditions which support colony formation amongst non-adherent cells produced in LTC generated from spleen of GM-CSF$^{-/-}$ mice,

| *Support medium*[a] [4] | *Frequency of CFC*[b] | | |
|---|---|---|---|
| | *DC colonies (Type I)*[c] | *DC colonies (Type II)*[c] | *Single DC*[d] |
| Nil | 0, 0, 0 | 1, 3, 4, | 0, 1, 2 |
| CSN-wild type LTC | 0, 0, 2 | 4, 5, 5 | 4, 6, 6 |
| CSN-mutant LTC | 0, 0, 0 | 3, 5, 5 | 1, 1, 2 |
| GM-CSF | 0, 0, 1 | 28, 30, 35 | 342, 399, 494 |
| KO Stroma | 2, 4, 4 | 12, 20, 21 | 459, 511, 570 |

a. CFC = colony forming cell number per $10^5$ cells plated in agar. Colonies are scored as clusters of 5 or more DC present after 14 days. The non-adherent cells population was collected from 14 week old spleen LTC derived from GM-CSF$^{-/-}$ mice. Data represent CFC in three replicates.

b. Assays were supplemented with either 50% CSN from LTC-X1 (wild type) or LTC generated from GM-CSF$^{-/-}$ mice (8FZ8.1), GM-CSF at 300U/ml or $10^4$ stromal cells derived from the GM- CSF$^{-/-}$ LTC (8FZ8.1).

c. DC colonies were identified morphologically. Type I colonies contain DC with obvious short or long membrane extensions. Type II colonies contain only large rounded cells with short membrane extensions. No other colony types were detected.

d. Single DC or clusters of less than 5 cells with veils or membrane extensions.

Colony assays using cells from GM-CSF$^{-/-}$ spleen LTC which were supplemented with stromal cells or GM-CSF alone, contained a large number (~4–500) of individual, viable, mature DC characterised by long membrane extensions (Table I). Both GM-CSF and stromal cells can individually support the survival but not the replication of fully developed DC in colony assays. Cells did not survive in assays supplemented with conditioned medium from either GM-CSF$^{-/-}$ or WT spleen LTC in the absence of stroma or GM-CSF. All data confirm the essential role of stromal cells in the production of DC in LTC consistent with previous studies which failed to detect production of GM-CSF in LTC (Ni & O'Neill, 1997). However, GM-CSF can act as a very effective alternative maintenance factor for mature DC survival. It also supports proliferation/differentiation of a small number of DC colonies from progenitors.

### DISCUSSION

Previously it was shown that cells resembling DC both functionally and phenotypically could be produced in LTC without the addition of an exogenous source of GM-CSF (Ni & O'Neill; 1997,1998,1999). This result contradicted many other reports on the importance of GM-CSF in production of DC *in vitro* from precursors isolated from blood, BM and peripheral lymphoid sites. A soluble biologically active factor resembling GM-CSF could not be detected in conditioned medium collected from LTC (Ni & O'Neill, 1997). However, it was not possible to rule out the presence of endogenously produced GM-CSF secreted in very low levels or produced and presented to cells via a cell bound receptor such as heparan sulphate (Roberts et al., 1988).

Data presented here relate to the importance of GM-CSF in DC development. While GM-CSF is not a critical factor in DC development in LTC, it can act as a maintenance factor to increase the survival of DC *in vitro*. Even as a maintenance factor it is not critical and stromal cells from GM-CSF$^{-/-}$ mice can act as an effective substitute support matrix and there may be other stroma produced factors which can maintain DC survival. Recent investigations have also confirmed the absence of *GM-CSF* gene transcription amongst the total cell population produced in LTC from wild type mice. This was determined using polymerase chain reaction amplification of cDNA prepared from a mixture of stromal and haemopoietic cells (Le & O'Neill, unpublished data).

LTC producing DC can be established from both BM and spleen cell populations of GM-CSF$^{-/-}$ mice. While stroma takes longer to form in GM-CSF$^{-/-}$LTC than in WT LTC, GM-CSF$^{-/-}$ spleen and BM LTC actively produce cells resembling DC after 16 weeks. Mutational loss of GM-CSF appears to impact on the nature and growth potential of the stromal cell layer. However, the effect is not critical and an adequate stromal cell layer does develop within 4 weeks of LTC establishment.

DC produced in established GM-CSF$^{-/-}$ spleen LTC resemble DC produced in WT LTC in terms of cell morphology, phenotype and antigen presenting capacity. The proportion of large DC at 16 weeks is slightly lower in GM-CSF$^{-/-}$ spleen LTC than in WT cultures. However, since both GM-CSF$^{-/-}$ and WT LTC produce lower numbers of DC than the well developed LTC-X1 culture derived from B10.A(2R) mice, this difference could reflect strain differences in DC production. Similar numbers of large DC are produced in both GM-CSF$^{-/-}$ and WT LTC. Consistent with previous studies, spleen LTC are easier to establish than BM LTC and they produce higher numbers of non-adherent DC (Ni & O'Neill, 1997).

The importance of stromal cells and stroma-derived factors in DC development from haemopoietic progenitors maintained in LTC has been demonstrated previously (Ni & O'Neill, 1998). A combination of conditioned medium from LTC and stromal cells very effectively supports the formation of mature DC colonies in agar colony assays (Wilson et al., 2000 a). However, the essential growth factors produced by stromal cells have not yet been characterised. Conditioned medium from LTC alone will not support survival of cells indefinitely. With the addition of stromal cells, regeneration of the DC population is easily achieved (Ni & O'Neill, 1998; Wilson et al., 2000 a). Furthermore, non-adherent cells removed

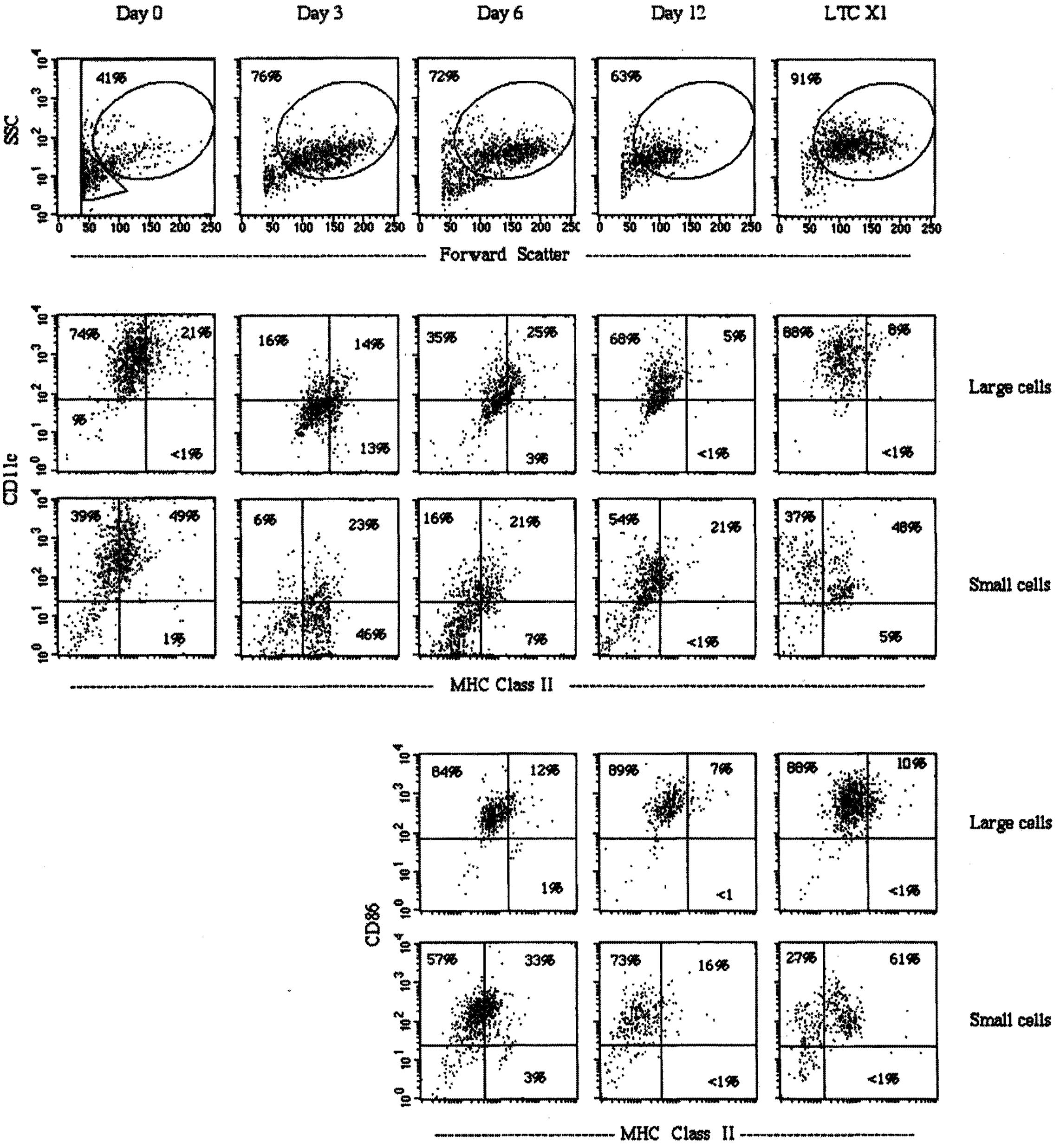

FIGURE 6 The effect of GM-CSF on development of DC in GM-CSF$^{-/-}$spleen LTC. Antigen expression and cell size distribution were monitored by FACS analysis on days 0, 3, 6 and 12 after addition of 300 U/ml of GM-CSF to the 16 week old 8FZ8.1. LTC. The production of large DC was monitored by Forward Scatter versus Side Scatter (FSC/SSC) analysis. The size of the large cell subset was identified by a gate imposed on the control LTC-X1 culture. Expression of MHCII, CD86 and CD11c was measured by antibody staining and FACS analysis on gated small and large LTC cells collected at different times after addition of GM-CSF to the culture. Again staining of LTC-X1 was used as a control from LTC and supported in medium supplemented with GM-CSF or GM-CSF in combination with interleukin(IL)-4, survive for only 1–2 weeks in the absence of stromal cells (data not shown). This has been demonstrated on many occasions for LTC derived from several different strains of mice. During culture with GM-CSF and IL-4, non-adherent cells collected from LTC adopt a more "dendritic-like" appearance with predominant membrane extensions. There is, however, no expansion of precursor cells and no new production of DC. Small cells disappear, cultures deteriorate after 1–2 weeks, and large DC die off (Ni & O'Neill, unpublished observations). GM-CSF and GM-CSF in combination with IL-4 are also ineffective in producing DC colonies from LTC-derived non-adherent cells in agar colony assays (Wilson et al., 2000 a). This again confirms an important role for stroma and stroma-produced soluble factors but not GM-CSF or IL-4 in the production of DC from progenitors.

GM-CSF can also substitute for stromal cells from GM-CSF$^{-/-}$ LTC in supporting the proliferation of a small number of DC colonies. This result is consistent with the presence of haemopoietic progenitors in LTC. Addition of GM-CSF to progenitors derived from GM-CSF$^{-/-}$ LTC induces differentiation/proliferation of colonies of DC with short membrane extensions. GM-CSF is not, however, a critical factor in the early stages of DC differentiation from progenitors and a small number of progenitors are present in LTC which are GM-CSF responsive. These may represent cells of a separate lineage or cells at a particular stage of development.

The data presented here also demonstrate a role for GM-CSF in maintenance or survival of mature DC *in vitro*. This was evident by an increase in the proportion of large cells present in GM-CSF$^{-/-}$ LTC supplemented with GM-CSF, and in agar colony assays where addition of GM-CSF maintained the survival of individual mature DC. The exact role of GM-CSF in the maintenance of DC survival is not yet clear but is under investigation. One possibility is that GM-CSF is anti-apoptotic and may act to increase the lifespan of DC as it does with eosinophils (Simon et al., 1997). Alternatively, it may influence the maturation or activation of DC at late stages during development. Cells from GM-CSF$^{-/-}$ mice may compensate for absence of a functional *GM-CSF* gene by utilising other stroma-derived factors to support DC survival. This argument is supported by evidence that addition of GM-CSF to LTC derived from GM-CSF$^{-/-}$ mice leads to an increase in the proportion of large DC present in LTC with no major changes in cell phenotype (Fig. 6). GM-CSF may initiate an additional pathway to support DC survival or maturation from precursors.

Another consideration is whether LTC-DC are more closely related to the apparent myeloid or lymphoid DC lineages (Shortman & Caux, 1997). Since LTC-DC develop from progenitors which grow independently of GM-CSF, they could be closely aligned with lymphoid DC. However, absence of CD8α expression and low expression of Dec-205 would suggest that LTC-DC are more likely myeloid DC. Cells described here originate from BM and spleen which could contain a mixture of progenitors specific for DC of several different lineages. In the absence of lineage-specific markers, it is difficult to relate the phenotypes of DC cultured *in vitro* to the phenotype of cell subsets present in different tissue sites *in vivo* and which may have different lineage origins (Salomon et al., 1998; Leenen et al., 1998). An alternative explanation is that LTC-DC are predominantly myeloid DC, but that myeloid DC have no critical requirement for GM-CSF during development. Addition of GM-CSF to cells in culture may, however, increase DC survival. Further analysis of DC subsets and their development both *in vivo* and within LTC will require the development of antibodies uniquely specific for cells of the different DC lineages.

### METHODS AND MATERIALS

### Animals

Mice were bred under specific pathogen-free conditions in the John Curtin School of Medical Research, Canberra, Australia and used when 6 to 10 weeks of age. GM-CSF$^{-/-}$ mice and GM-CSF$^{+/+}$ littermate controls [(129 × C57BL/6J) $F_1$ hybrid] were purchased from the Ludwig Institute for Cancer Research, Melbourne, and used at 10 weeks of age.

### Production of dendritic cells in LTC

LTC were established from spleen and bone marrow (BM) as previously described (Ni & O'Neill, 1997; 1998; 1999). Single cell suspensions were made by pressing spleen or BM through a fine wire mesh. Cells were cultured at 37°C in plastic tissue culture flasks (Nunclon, Roskilde, Denmark) at a concentration of 2.5 × $10^6$ cells/ml in an atmosphere of 5% $CO_2$ in air. Dulbecco's modified Eagle's medium (Gibco, Gaitherburg, MD) was used for cell culture following supplementation with 10% fetal calf serum (FCS), 5 × $10^{-5}$M 2-mercaptoethanol, 10 mM Hepes, 100 U/ml penicillin, 100 μg/ml streptomycin, 4g/L glucose, 6 mg/L folic acid, 36 mg/L L-asparagine, 116 mg/L L-arginine HCl (sDMEM).

Cultures were initially left undisturbed for 10–14 days after establishment. Medium change involved replacement of 75% volume every 5 days or when cultures became acidic. A stromal cell layer of fibroblasts and endothelial cells formed initially. By 4 weeks, lymphoid and myeloid cells disappeared from the culture and foci of haemopoietic cells began to appear on stroma. By 6–8 weeks these began to shed non-adherent cells into the supernatent which were collected for experimentation. Data has been obtained here from multiple cultures from many individual mice and specific analysis has been performed on two different spleen LTC, 8GS1.4 and 8FZ8.1.

The LTC-X1/3 spleen cultures were established from B10.A(2R) mice (Ni & O'Neill, 1997). It has been maintained in continuous culture for ~6 years by passage of cells and stroma every 2–3 months. DC collected from LTC-X1 were used as controls in all experiments. GM-CSF was added to some cultures at 300 U/ml. This was purchased as recombinant mouse protein from Genzyme (Cambridge, MA). Secondary cultures were established by seeding BM cells on to LTC-X1 stroma which had been stripped of non-adherent cells and irradiated (2Gy; γ-source)

### Antibodies and fluorochromes

Antibodies used included FITC-conjugated anti-mouse I-$A^b$ (clone AF6–120.1); biotinylated anti-mouse CD11c (clone HL1), biotinylated anti-mouse CD86 (clone GL1), and purified anti-mouse CD32/CD64 (clone 2.4G2) (all from Pharmingen, San Diego, CA). Some antibodies were prepared as hybridoma supernatant. These included M1/70 (CD11b), AT83 (Thy-1.2), RA3–6B2 (B220), RB6–8C5 (Gr-1), F4/80 (macrophages/DC), NLDC-145(Dec-205), and 33D1 (spleen DC). Second stage reagents included goat anti-rat IgG $F(ab')_2$ (Silenus, Hawthorn, Victoria, Australia) and Streptavidin-phycoerytherin (PE) (Pharmingen, San Diego, CA).

### Single colour immunostaining

Antigen expression was assessed in either a direct assay using fluorochrome conjugated antibody or an indirect assay using a fluorochrome-conjugated second stage reagent. The staining procedure has been described previously (O'Neill & Ni, 1993). Briefly, non-adherent cells ($10^5$ – $10^6$) were collected from LTC, and washed twice with sDMEM/0.1% $NaN_3$by centrifugation at 700 g for 5 min. Specific antibody was absorbed to cells in a 50 μl volume in the wells of a microtitre plate. After incubation for 30 min on ice, cells were washed thrice with medium by centrifugation (350 g for 2 min). The same procedure was repeated for absorption of a labelled second stage reagent. Fluorescence was measured by log analysis using a FACSort (Becton Dickinson, San Jose, CA). For indirect staining, an isotype control antibody was used to indicate nonspecific staining levels and to set gates for FACS analysis. Incubation for 15 min at 4 °C with the 2.4G2 antibody was used to block Fc receptor binding of antibody (10 μl of 0.5 mg/ml 2.4G2).

For two colour staining, cells were incubated with 2.4G2 for 15 min at 4°C to block Fc receptor binding and then both FITC-conjugated and biotin-conjugated specific antibodies were added and cells incubated for 30 min on ice. Cells were washed twice with 5%

FCS/sDMEM/0.1% $NaN_3$ and once with PBS/0.1% $NaN_3$. Cells were then incubated with a fluorochrome-labelled second stage reagent for 30 min at 4°C to visualise antibody binding. Cells were washed thrice with PBS/0.1% $NaN_3$ prior to FACS analysis. Fluorescence was measured with a FACSort (Becton Dickinson, San Jose, CA), using an excitation wavelength of 448 nm. Cell populations of interest were gated and analysed using CellQuest™ software (Becton Dickinson, San Jose, CA). Isotype control antibodies were used to delineate positive staining above background.

### Mixed lymphocyte reaction (MLR)

Assays were carried out as described previously (O'Neill et al., 1999a). Non-adherent cells were collected from LTC, washed twice with sDMEM and then irradiated (20 Greys, γ-irradiation, $^{60}Co$ source) before use as antigen presenting cells (APC). They were added in graded numbers to $10^5$ spleen mononuclear cells as responders in a final volume of 200 μl in 96 well U-bottom microtitre plates. Responder spleen mononuclear cells were prepared by centrifugation through an Isopaque-Ficoll gradient (10 min, 25,000 rpm). Proliferation was measured by uptake of $^3$H-Thymidine ($^3$HT) over the final 6 h of a 72 h culture. Triplicate culture wells were labelled with 1μCi of $^3$HT label per well.

### In vitro agar colony assay

The method for colony assays has been previously described (Wilson et al., 2000 a). Non-adherent cells were collected from spleen LTC derived from GM-CSF$^{-/-}$mice, washed with sDMEM and resuspended into sDMEM at 37°C. Double-strength culture medium (1 ml of 2X sDMEM) at 37°C was mixed with Seaplaque agarose (1.0 ml of 1.6%w/v) (FMC Bioproducts, Rockland, ME) at 45°C and 2 ml of growth medium containing added factors at 37°C before the addition of 100 μl of cells at 2x$10^6$ cells/ml. One ml of the cell suspension was pipetted into replicate 35 mm gridded petri dishes (Nunclon, Roskilde, Denmark). After mixing to distribute cells, cultures were allowed to gel by cooling for 4–5 mins at 4°C. Cultures were then incubated at 37°C in 5% $CO_2$in air. Supplements to growth medium included (1.) GM-CSF, (2.) conditioned medium (CSN) collected from LTC or (3.) stromal cells prepared from LTC 8FZ8.1, derived from the spleen of GM-CSF$^{-/-}$ mouse. CSN was collected from LTC-X1 or from spleen LTC established from GM-CSF$^{-/-}$ mice at 48 and 72 h after medium change.

After 7,10 and 14 days of incubation, colony development was monitored microscopically. At 14 days, agar cultures were fixed with acetone for *in situ* Wright-Giemsa staining. DC can be identified by their unique staining pattern with Wright-Giemsa. Discrete aggregates of 5 or more DC were scored as colonies, in line with other published reports (Reid et al., 1992). DC cells were identified by their membrane extensions and characteristic staining of dark blue nucleus and faint pink cytoplasm. DC staining is very distinctive in comparison with other cell types including monocytes/macrophages (unpublished data). Type I DC colonies were identified by short membrane extensions on cells and Type II DC colonies by long membrane extensions.

### *Acknowledgements*

The authors gratefully acknowledge the assistance of A Dunn, Ludwig Institute for Cancer Research, Melbourne, who kindly provided knockout mice for this study. This work was supported by grants to HO from the National Health and Medical Research Council, the Australian Research Council and the Clive and Vera Ramaciotti Foundation.

### *References*

Bjorck, P., and Kincade, P.W. (1998) CD19+ pro-B cells can give rise to dendritic cells in vitro. J Immunol **161**: 5795–9.

Caux, C., and Banchereau, J. (1996) in Whetton, A., and Gordon, J. (Eds.) Blood cell Biochemistry Vol **7**, "Haemopoietic Growth Factors and their Receptors." Plenum Press, London.

Leenen, P.J., Radosevic, K., Voerman, J.S., Salomon, B., van Rooijen, N., Klatzmann, D., van Ewijk, W. (1998) Heterogeneity of mouse spleen dendritic cells: in vivo phagocytic activity, expression of macrophage markers, and subpopulation turnover. J Immunol **160**: 2166–73.

Maraskovsky, E., Brasel, K., Teepe, M., Roux, E.R., Lyman, S.D., Shortman, K., and McKenna, H.J. (1996) Dramatic increase in the numbers of functionally mature dendritic cells in Flt3 ligand-treated mice: multiple dendritic cell subpopulations identified. J Exp Med 1996 **184**: 1953–62.

Ni K., and O'Neill H.C. (1997) Establishment of long term stromal cultures producing dendritic cells. British Journal of Haematology **97**, 710–725.

Ni, K. and O'Neill, H.C. (1998) Haemopoiesis in long term stroma-dependent cultures from lymphoid tissues: Production of cells with myeloid/dendritic characteristics. In vitro Cellular and Molecular Biology **34**: 298–307.

Ni K., and O'Neill H.C. (1999) Spleen stromal cells support haemopoiesis and in vitro growth of dendritic cells from bone marrow. British Journal of Haematology, **105**: 58–67.

Ni, K., and O'Neill, H.C. (2000) Improved FACS analysis confirms generation of dendritic cells in long-term stromal dependent spleen cultures. Immunology & Cell Biology **78**: 196–204.

O'Neill H.C., Ni K., and Wilson, H. (1999a) Long-term stroma dependent cultures are a consistent source of immunostimulatory dendritic cells. Immunology & Cell Biology **77**: 434–441.

O'Neill H.C., Jonas N., Wilson H., and Ni, K. (1999b) Immunotherapeutic potential of dendritic cells generated in long term stroma-dependent cultures. Cancer Biotherapy and Radiopharmaceuticals **14**: 263–76.

Pierre, P., Turley, S.J., Gatti, E., Hull, M., Meltzer, J., Mirza, A., Inaba, K., Steinman, R.M., and Mellman, I. (1997) Developmental regulation of MHC class II transport in mouse dendritic cells. Nature **388**: 787–92.

Reid, C.D., Stackpoole, A., Meager, A., and Tikerpae, J. (1992) Interactions of tumor necrosis factor with granulocyte-macrophage colony-stimulating factor and other cytokines in the regulation of dendritic cell growth in vitro from early bipotent CD34+ progenitors in human bone marrow. J Immunol **149**: 2681–8.

Roberts, R., Gallagher, J., Spooncer, E., Allen, T.D., Bloomfield, F., and Dexter, T.M (1988) Heparan sulphate bound growth factors: a mechanism for stromal cell mediated haemopoiesis. Nature **332**: 376–8.

Salomon, B., Cohen, J.L., Masurier, C., and Klatzmann, D. (1998) Three populations of mouse lymph node dendritic cells with different origins and dynamics. J Immunol **160**: 708–17.

Saunders, D., Lucas, K., Ismaili, J., Wu, L., Maraskovsky, E., Dunn, A., and Shortman, K. (1996) Dendritic cell development in culture from thymic precursor cells in the absence of granulocyte/macrophage colony-stimulating factor. J Exp Med **184**: 2185–96.

Shortman, K., and Caux, C. (1997) Dendritic cell development: multiple pathways to nature's adjuvants. Stem Cells **15**: 409–19.

Simon, H.U., Yousefi, S., Dibbert, B., Levi-Schaffer, F., and Blaser, K. (1997) Anti-apoptotic signals of granulocyte-macrophage colony-stimulating factor are transduced via Jak2 tyrosine kinase in eosinophils. Eur J Immunol 1997 **27**: 3536–9.

Vremec, D., Lieschke, G.J., Dunn, A., Robb, L., Metcalf, D., and Shortman, K. (1997) The influence of granulocyte/macrophage colony stimulating factor on dendritic cell levels in mouse lymphoid organs. European Journal of Immunology **27**: 40–44.

Wilson H., Ni K., and O'Neill H.C. (2000 a) Continuous production of immature dendritic cells from progenitors maintained in a long term spleen stromal culture. Experimental Haematology **28**: 193–202.

Wilson, H., Ni, K., and O'Neill, H.C. (2000 b) Identification of progenitor cells in long term stromal cultures which produce immature dendritic cells. Proc Natl Acad Sci USA **97**: 4784–9.

